# Identification of dietary alanine toxicity and trafficking dysfunction in a *Drosophila* model of hereditary sensory and autonomic neuropathy type 1

**DOI:** 10.1093/hmg/ddv390

**Published:** 2015-09-22

**Authors:** Matthew C. W. Oswald, Ryan J. H. West, Emyr Lloyd-Evans, Sean T. Sweeney

**Affiliations:** 1 Department of Biology and Hull-York MedicalSchool, University of York, YorkYO10 5DD, UK and; 2 School of Biosciences, Cardiff University, Museum Avenue, Cardiff CF10 3AX, UK

## Abstract

Hereditary sensory and autonomic neuropathy type 1 (HSAN1) is characterized by a loss of distal peripheral sensory and motorneuronal function, neuropathic pain and tissue necrosis. The most common cause of HSAN1 is due to dominant mutations in serine palmitoyl-transferase subunit 1 (SPT1). SPT catalyses the condensation of serine with palmitoyl-CoA, the initial step in sphingolipid biogenesis. Identified mutations in *SPT1* are known to both reduce sphingolipid synthesis and generate catalytic promiscuity, incorporating alanine or glycine into the precursor sphingolipid to generate a deoxysphingoid base (DSB). Why either loss of function in *SPT1*, or generation of DSBs should generate deficits in distal sensory function remains unclear. To address these questions, we generated a *Drosophila* model of HSAN1. Expression of *dSpt1* bearing a disease-related mutation induced morphological deficits in synapse growth at the larval neuromuscular junction consistent with a dominant-negative action. Expression of mutant *dSpt1* globally was found to be mildly toxic, but was completely toxic when the diet was supplemented with alanine, when DSBs were observed in abundance. Expression of mutant *dSpt1* in sensory neurons generated developmental deficits in dendritic arborization with concomitant sensory deficits. A membrane trafficking defect was observed in soma of sensory neurons expressing mutant *dSpt1*, consistent with endoplasmic reticulum (ER) to Golgi block. We found that we could rescue sensory function in neurons expressing mutant *dSpt1* by co-expressing an effector of ER–Golgi function, Rab1 suggesting compromised ER function in HSAN1 affected dendritic neurons. Our *Drosophila* model identifies a novel strategy to explore the pathological mechanisms of HSAN1.

## Introduction

Hereditary sensory and autonomic neuropathy type 1 (HSAN1) is a rare disorder pathologically characterized by distal sensory loss and peripheral ulceration, predominantly in lower limbs (1–5). Degeneration of motorneurons is also known to occur in addition to distal limb weakness and muscle atrophy (4). The condition is caused in the majority of cases by inheritable dominant mutations in the genes encoding subunits of the enzyme serine palmitoyl-transferase (SPT). SPT function is essential for catalysis of the first step in *de novo* sphingolipid synthesis at the ER (6,7). SPT exists as a heterodimer of homologous subunits, SPT long chain base subunit 1 (SPTLC1/SPT1) and SPT long chain base subunit 2 (SPTLC2/SPT2), that catalyse the pyridoxal phosphate-dependent condensation of l-serine and palmitoyl-coenzyme-A to generate 3-ketosphinganine (8). A third subunit present in mammals, SPTLC3 contributes to the generation of short C16 sphingoid bases (9). At present, the manner in which the dominant mutations in SPT1 affect neuronal function, particularly in peripheral nerves, is unclear.

A number of HSAN1-related missense mutations (C133W, C133Y and V144D) have been identified in SPT1 from 24 affected families (6,7) of which C133W appears to be the most common (7). More recently, mutations in SPT2 that cause a form of HSAN1 have been identified (10,11). A structural determination of the homologous prokaryotic enzyme, which exists as a homodimer, has given some insight into how disease mutations may affect the activity of the enzyme (8,12). Initially, reports suggested that disease-related mutations in SPT1 generated a gain-of-function mechanism in SPT resulting in increased cellular glucosylceramide levels (7). Others suggested a dominant-negative mechanism, albeit a mechanism of selective or reduced sphingolipid synthesis while overall sphingolipid levels were maintained (13,14). Recent data, however, have suggested that the SPT1 disease causing mutations may be neomorphic and cause a shift in substrate specificity from serine to alanine or glycine incorporation (15,16). This in turn generates atypical unmodifiable deoxysphingoid bases (DSBs) which, based on their *in vitro* activity, are proposed to be neurotoxic (15,16). Whether the mutations act in a predominantly neomorphic (production of toxic DSBs) or dominant-negative (reduction of sphingolipid synthesis) manner, or if the two mechanisms overlap to generate pathology remain unclear. Why peripheral neurons are predominantly affected by mutations in this enzyme similarly remains unexplained.

To understand the pathological function of HSAN1 mutations in SPT1, and the possible toxic effect of dietary alanine *in vivo*, we have employed a transgenic approach using the genetically tractable *Drosophila* larval peripheral nervous system as a model system. Using a novel nociception assay (17), selective enrichment of amino acids in the diet and examination of membrane traffic in sensory neurons, we determined a direct relationship between the disease mutations, dietary alanine, the presence of DSBs and sensory function. Additionally we identify a potential pathological mechanism of disrupted ER to Golgi traffic in these neurons. Our findings suggest a novel mechanism for sensory dysfunction that is consistent with deficits in distal peripheral neurons and further highlight a critical functional role for the secretory system in peripheral dendritic processes.

## Results

### *dSpt1* is an essential gene in *Drosophila*

**Figure 1.**
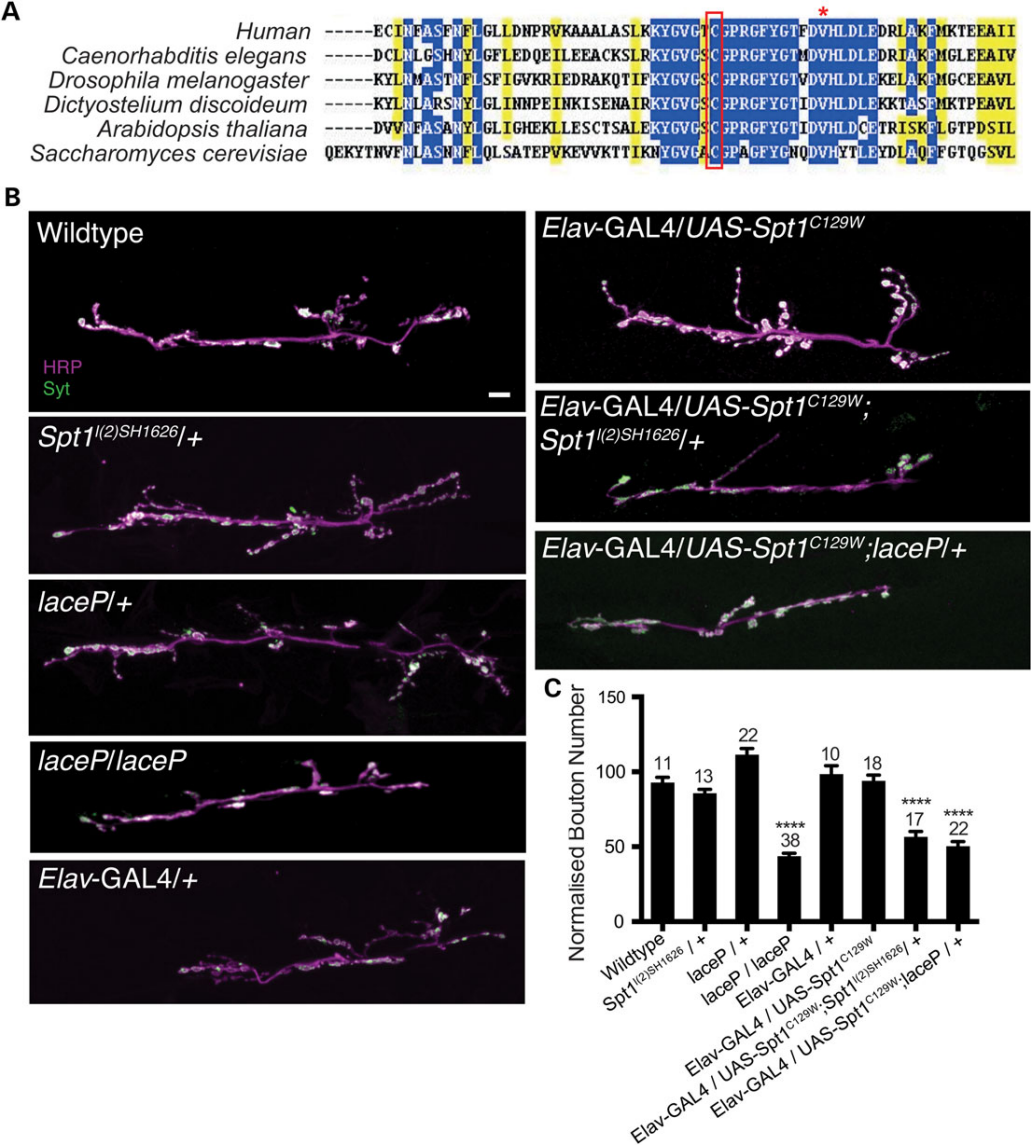
*Spt1^C129W^* acts as a genetic dominant negative to alter synaptic growth parameters. (**A**) C133W/Y are the most common HSAN1-causing mutations. C133 and its fly orthologue C129 are highly conserved and lie within a highly conserved protein domain. Accession numbers: *Homo sapiens* AAH68537, *Drosophila melanogaster* AAM68597, *Caenorhabditis elegans* P91079, *Dictyostelium discoideum* Q55FL5.1, *Arabidopsis thalania* CAB16844 and *Saccharomyces cerevisiae* AAA34739. Red asterisk indicates the site of V144D mutations that also give rise to HSAN1. (**B**) Global disruption of Spt1 function using mutants of *Spt2/lace* and neuronal expression (*Elav*-GAL4) of UAS-*Spt1^C129W^* transgenes, in combination with an *Spt1* mutant (*Spt1^l(2)SH1626^*), disrupt NMJ growth parameters at the 6/7 muscle, hemisegment A3. Scale bar = 10 µm. (**C**) Quantification of growth parameters at neuromuscular synapse 6/7 hemisegment A3. ****P ≤ 0.0001.

HSAN1 is caused by mutations in the *SPTLC1* gene, the most common mutation being C133W (6,7). The *SPTLC1* gene encodes SPT1, which localizes to the ER membrane and associates with cytosolic SPT2 to form the type 1 ER protein SPT (18). SPT performs the initial step in *de novo* sphingolipid synthesis, catalysing the condensation of l-serine and palmitoyl-coA to form ketosphinganine. Position C133 shows a high degree of evolutionary conservation (Fig. 1A) and lies within a highly conserved protein domain that has been linked to critical roles in SPT activity and substrate specificity (8).

To aid our analysis, we identified alleles of *Drosophila dSpt1*. From the *Drosophila* genome project and Bloomington stock centre, we identified a *P*-element insertion in the 5′ regulatory region of *dSpt1, dSpt1^l(2)SH1626^* (19). When crossed to a deficiency stock uncovering the region, *Df(2)vg-B*, we found that development of the *P*-element/deficiency transheterozygotes was delayed by 3–5 days. We used this delayed eclosion phenotype to screen previously identified but unsequenced point mutations from the genomic region in *dSpt1*. We identified two point mutations *l(2)49Fb^1^* and *l(2)49Fb^4^* in the *dSpt1* locus (20). Sequencing of the *dSpt1* locus in *l(2)49Fb^1^* allele revealed a C251Y nonsense mutation, while sequencing of the *l(2)49Fb^4^* allele uncovered a Q90–STOP truncation. Furthermore, we used a *P*-element imprecise excision strategy to delete 1.6 kb (including the start codon) of the *dSpt1* locus and generate the *dSpt1^JO8^* allele (Supplementary Material, Fig. S1). When the *dSpt1^l(2)SH1626^* mutant was crossed to the *l(2)49Fb^4^*, *l(2)49Fb^1^* or *dSpt1^JO8^* alleles, all mutant combinations were found to have the 3–5 day delayed development phenotype. Complementation crosses of *l(2)49Fb^4^*, *l(2)49Fb^1^* and *dSpt1^JO8^* to each other were lethal at first instar, confirming our *dSpt1^l(2)SH1626^* allele as an *dSpt1* hypomorph. Global expression of the UAS-*dSpt1* transgene under the control of *tubulin*-GAL4 was also sufficient to rescue all transheterozygous *dSpt1* mutant combinations, allowing survival to adulthood. Neuronal (*Elav*-GAL4) or muscle (*MHC*-GAL4) driven expression of UAS-*dSpt1* failed to rescue *dSpt1* mutants to adulthood. We can therefore conclude that the *dSpt1* locus, like the *dSpt2* locus (*lace*) (21), is an essential gene in *Drosophila* and required globally.

### *dSpt1^C129W^* acts in a dominant-negative manner at the *Drosophila* larval neuromuscular synapse to alter synapse growth parameters

HSAN1 patients have peripheral- and motorneuronal functional deficits (4). To try to dissect the mechanism driving SPT1^C133W^ dysfunction in motor neurons, we examined the effects of expression of a mutated *Drosophila* SPT1 protein at the larval neuromuscular synapse.

**Figure 2.**
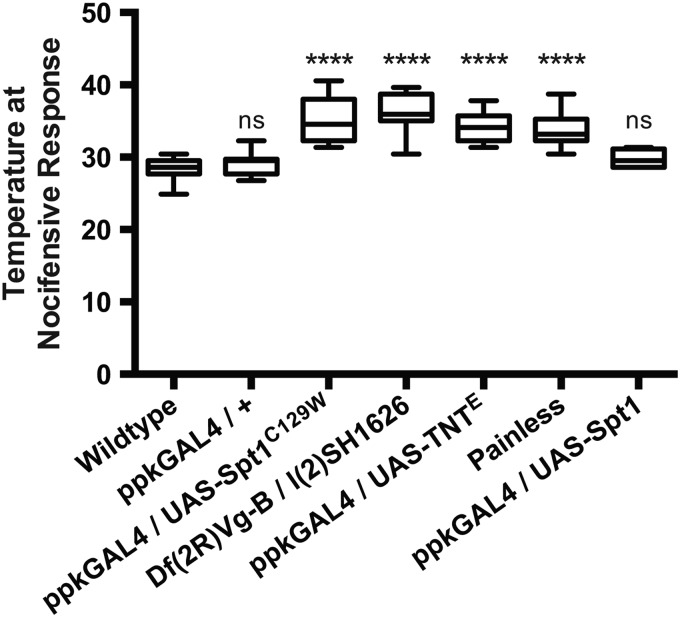
Spt1 function is required for appropriate larval nociceptive responses to rising heat. Targeted *Spt1^C129W^* expression under control of the *ppk*-GAL4 driver in the class IV da neurons, and global hypomorphic conditions of *Spt1* causes defective nociception similar to that observed during da neuron silencing (*ppk*-GAL4 driving expression of UAS-TNT^E^) and in the *painless* (*pain^1^*) mutant. ****P ≤ 0.0001, ANOVA, ns = non-significant compared with control.

We obtained the cDNA of the *Drosophila SPT1* (*dSpt1*), introduced a C to W substitution at amino acid position 129 (analogous to the human 133, Fig. 1A) by site-directed-mutagenesis and subcloned both the wild-type and mutant subunit into the pUASt vector (22). We generated multiple independent UAS-*dSpt1* and UAS-*dSpt1^C129W^* transgenes by microinjection, allowing us to express both forms in a tissue-specific manner under the control of the GAL4 transcriptional activator. When expressed with *ppk*-GAL4, all UAS-*dSpt1*-C129W transgenes generated a defective nociception response (see Fig. 2), while expression of UAS-*dSpt1* gave responses no different to wild-type animals.

To ascertain the effect of dSpt1^C129W^ expression on neuronal growth, we expressed the dSpt1^C129W^ protein in the nervous system of the developing *Drosophila* larva using the pan-neuronal *Elav*-GAL4 driver and examined the effect on third instar neuromuscular junction (NMJ) morphology (Fig. 1B and C). The *Drosophila* larval NMJ is a genetically tractable and accessible model glutamatergic synapse that is well characterized for growth and morphology (23,24). Using homozygotes of a previously isolated hypomorphic allele of dSpt2, *dSpt2/lace^P^* (21) with a sphingolipid content known to be 2.58% of wild-type levels (25) we determined that a loss of SPT activity generated a reduction in synaptic bouton number, reducing bouton number by 50%.

Pan-neuronal (*Elav*-GAL4) expression of UAS-*dSpt1^C129W^* resulted in no change in synaptic bouton number at the larval muscle 6/7, hemisegment A3 NMJ (Fig. 1B and C). Introduction of heterozygous alleles of *dSpt1*, or *dSpt2*/*lace* in the presence of globally expressed *dSpt1^C129W^*-induced lethality at pupal stage. Introduction of heterozygous alleles of *dSpt1* or *dSpt2*/*lace^P^* in the presence of the neuronally expressed *dSpt1^C129W^* induced a reduction of bouton number of 50%, phenocopying the homozygous loss-of-function allele of *dSpt2*/*lace^P^* (Fig. 1B and C). This genetic interaction between *dSpt1^C129W^* expression and heterozygous mutations of *dSpt1* and *dSpt2*/*lace^P^* strongly suggests a dominant-negative function for the *dSpt1^C129W^* mutation when examined in the context of lethality and synaptic growth.

### *dSpt1^C129W^* expression in sensory neurons of *Drosophila* larvae induces a sensory deficit

HSAN1 manifests as distal sensory loss, allowing recurrent and progressive tissue damage due to injury. We probed our *Drosophila* model of HSAN1 for any effect in the response to noxious heat stimuli. It has been shown previously that in response to noxious heat thresholds, third instar larvae perform a rapid and characteristic barrel-rolling escape behaviour. This behavioural response was previously used to identify the *painless* mutant (28). We recently developed a novel thermosensation/nociception assay based on this rolling behaviour elicited by excessive heat in a droplet of water and used it to ask whether third instar larvae expressing *dSpt1^C129W^* in the class IV da neurons show a defective noxious heat response (17). Expression of *dSpt1^C129W^* significantly increased the nociceptive response temperature (Fig. 2), suggesting that *dSpt1^C129W^* expression in the class IV da neurons is sufficient to perturb nociception. The *painless* mutant and the expression of tetanus-toxin light chain in the class IV da neurons show a similar delayed response. The expression of tetanus toxin light chain, shown previously to promote synapse silencing (29), produces a shift in nociceptive response temperature but like the *pain^1^* mutant and the *dSpt1^C129W^* manipulation does not remove it completely; most likely due to the presence of other thermosensors tuned to proximate temperature ranges in the peripheral nervous system (30). These data reveal that the class IV da neurons contribute significantly, but not exclusively, to the larval response to heat, and that *dSpt1^C129W^* expression in class IV da neurons compromises this response while *dSpt1* expression produces a response indistinguishable from wild-type animals. To probe the functional integrity of the central synapses of the Class IV sensory neurons in larvae, we co-expressed a channelrhodopsin construct (31) with *dSpt1^C129W^* using the *ppk*-GAL4 driver (32). Light activation of the channelrhodopsin elicited a nocifensive escape response in the presence of *dSpt1^C129W^* suggesting that the synapses of the class IV sensory neurons are still able to function (Supplementary Material, Movie S1). We examined the morphology of the synapses of the Class IV dendritic neurons in the larval ventral nerve cord when either *dSpt1* or *dSpt1^C129W^* are expressed with the *ppk*-GAL4 driver (Supplementary Material, Fig. S2). We observed no gross morphological differences in synapse structure between *dSpt1*, *dSpt1^C129W^* expressing animals and wild-type. We conclude from this series of experiments that the sensory dendrite function of class IV da neurons are likely to be compromised by *dSpt1^C129W^* expression.

### In the presence of *dSpt1^C129W^*, enrichment of alanine in the larval diet is toxic and can be partially alleviated by increasing serine dietary abundance

The discovery of possible substrate promiscuity in the SPT enzyme complexes bearing HSAN1 mutations (15,16) proposed a novel, neomorphic mechanism for *dSpt1^C129W^* toxicity. Cysteine 133 of SPT1 associates intimately with lysine 265 in SPT2 upon heterodimer assembly. It is proposed that the substitution of a large tryptophan residue compromises SPT2 lysine 265 function and perturbs Schiff base association with the SPT co-factor pyridoxal-5-phosphate. The occlusion of the active site is proposed to allow the entry of smaller amino acids such as alanine and glycine in preference to serine (15,16). Addition of alanine or glycine to palmitoyl-CoA generates a deoxyspingoid base that cannot be further modified to generate normally biologically active and diverse sphingolipid species. This finding suggests that an enrichment of alanine or glycine in the diet of larvae expressing the dSpt1^C129W^ protein should be toxic.

**Figure 3.**
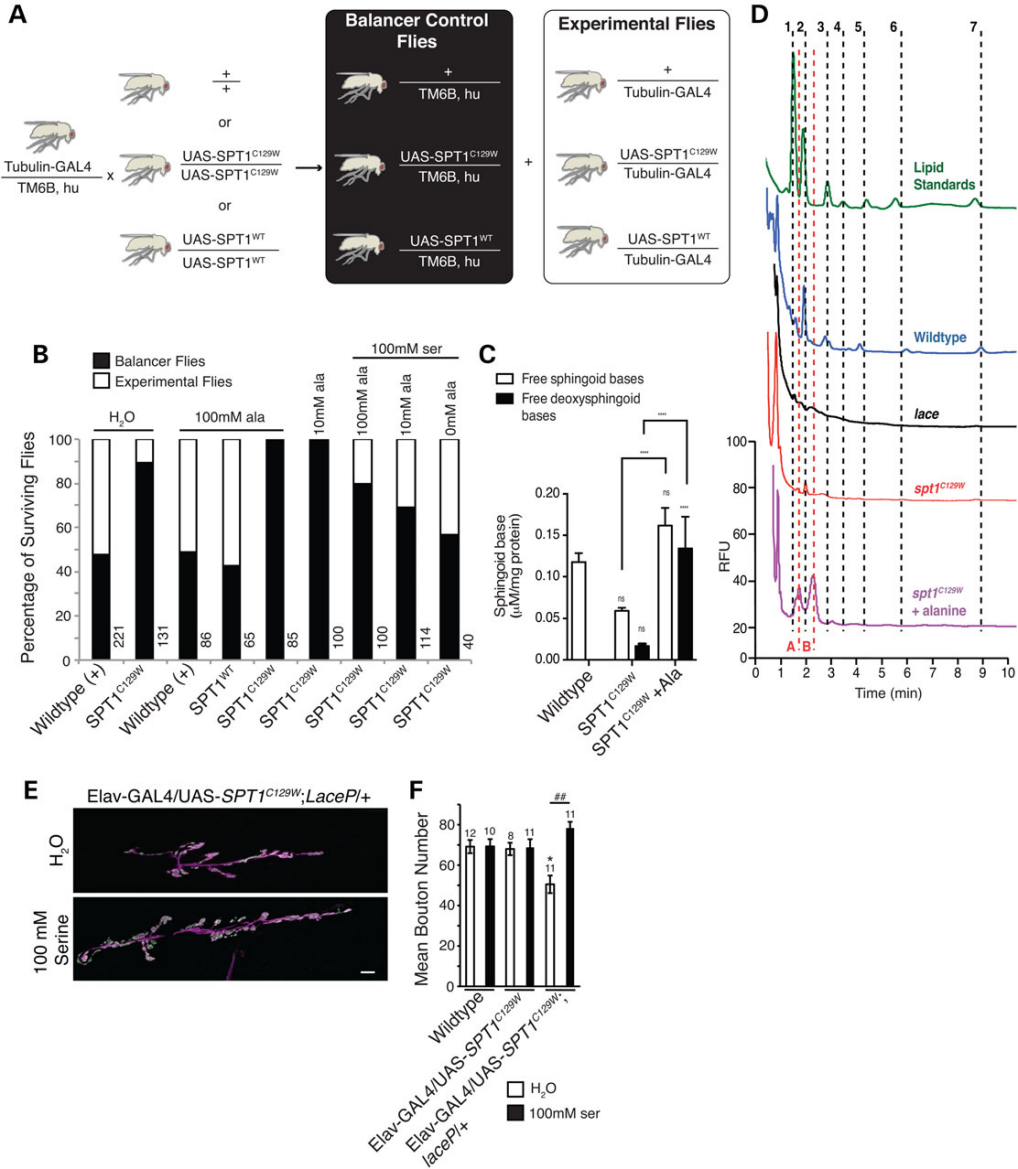
Feeding alanine to *Spt1^C129W^* expressing flies induces lethality and the appearance of DSBs. (**A**) Schematic representation of crossing scheme used to produce *Spt1^C129W^* expressing flies and control sibling flies lacking the *Spt1^C129W^* transgene for feeding experiments. (**B**) Feeding of alanine and serine to flies expressing *Spt1^C129W^* or wild-type *Spt1*. White boxes represent percentage experimental flies surviving, black boxes sibling controls surviving. (**C**) Quantification of total free endogenous sphingoid base (C14, C16 and C18 sphingosine and sphinganine) and free DSB (C14 deoxysphingosine and deoxysphinganine) content of *Drosophila* by HPLC analysis. ****P ≤ 0.0001, two-way ANOVA with Tukey's multiple comparison test. (**D**) Representative HPLC traces illustrating identification of each peak in the unknown samples based on known standards (green trace), confirmation of peaks representing endogenous sphingoid bases using the sphingoid base null *lace* mutants (black trace) or confirmation of the presence of DSBs via the alanine fed *spt1^C129W^* expressing animals (purple trace). Numbers correspond to endogenous free sphingoid bases, 1 and 2 = C14 chain length sphingosine and sphinganine respectively; 3 and 4 = C16 sphingosine and sphinganine, respectively; 5 and 6 = C18 sphingosine and sphinganine, respectively. A and B correspond to deoxysphingosine and deoxysphinganine respectively. For all experimental conditions *n* = 5–8, apart from *lace* where *n* = 2. ns = non-significant compared with control. (**E**, **F**). Disruption of larval synaptic growth parameters at the 6/7 muscle, hemisegment A3 in animals of the genotype *Elav*-GAL4/UAS-*Spt1^C129W^*, *lace^P^* can be rescued by the feeding of serine. Scale bar = 10 µm, ANOVA: *F* (d.f. 5) = 4.4; *P* < 0.01, Dunnett’s post hoc comparison to WT (*P < 0.05) and Bonferroni comparison between groups (^##^*P* < 0.01).

We raised larvae globally expressing either wild-type *dSpt1* or *dSpt1^C129W^* on diets containing 10 mm or 100 mm alanine. We found enriched alanine to be 100% toxic to larvae expressing *dSpt1^C129W^*, but not animals expressing wild-type *dSpt1*, control wild-type animals or siblings bearing a balancer chromosome (and therefore lacking the tubulin-GAL4 element to drive expression of *dSpt1* or *dSpt1^C129W^*) (Fig. 3A and B). Toxicity of alanine for animals expressing *dSpt1^C129W^* protein could be partially alleviated by co-feeding 100 mm serine. Serine feeding had no effect on animals expressing either the wild-type dSpt1 or dSpt1^C129W^ protein in the absence of alanine supplementation. On normal lab yeast/agar food, survival for larvae expressing *dSpt1^C129W^* is unaffected (data not shown). Raising larvae on ‘instant’ laboratory food reduces survival for *dSpt1^C129W^* expressing larvae suggesting a high content of alanine or glycine (Fig. 3B). Supplementing ‘instant’ food with 100 mm serine brings survival of *dSpt1^C129W^* expressing larvae to wild-type levels. Our data support the findings that alanine is toxic in the presence of an HSAN1 mutation in SPT1.

### Global expression of *dSpt1^C129W^* generates a reduction of normal sphingolipids, and upon feeding of alanine reveals the presence of DSBs

We interrogated our model of HSAN1 by globally expressing *dSpt1^C129W^* and assessed the abundance of sphingolipids. As can be seen in the graph (Fig. 3C and D), total sphingoid base abundance is reduced in animals globally expressing *dSpt1^C129W^* compared with controls, while the content of DSBs is only marginally elevated in the *dSpt1^C129W^* animals grown on normal lab food (with no DSBs found in the wild-type animals), suggesting a dominant-negative effect of this mutation on sphingolipid biosynthesis. However, following feeding with 100 mm alanine, total sphingoid base content in the *dSpt1^C129W^* expressing animals is restored back to untreated control levels but is coupled to an ∼8-fold elevation in the free DSB content compared with the animals grown on normal lab food, confirming a neomorphic effect following the change in diet. Confirmation of the identity of each sphingoid base analysed by HPLC is shown in Fig. 3D. Alanine fed animals globally expressing *dSpt1^C129W^* were used to confirm the identity of the DSB peaks and homozygous *dSpt2*/*lace^P^* hypomorphs were used as positive controls to identify endogenous sphingoid bases in the wild-type traces based on their absence in *dSpt2*/*lace^P^* samples (previously shown to have a sphingolipid content of 2.58% (25)). Interestingly, feeding of serine can rescue the synaptic development phenotype in an *Elav*-GAL4, UAS-*dSpt1-C129W*, *lace^P^/+* animal (Fig. 3E and F). These data confirm that *dSpt1^C129W^* over-expression in *Drosophila* acts in a manner similar to that identified in the mammalian system (15,16).

### Secretory and membrane dysfunction is observed in sensory neurons expressing *dSpt1^C129W^*

**Figure 4.**
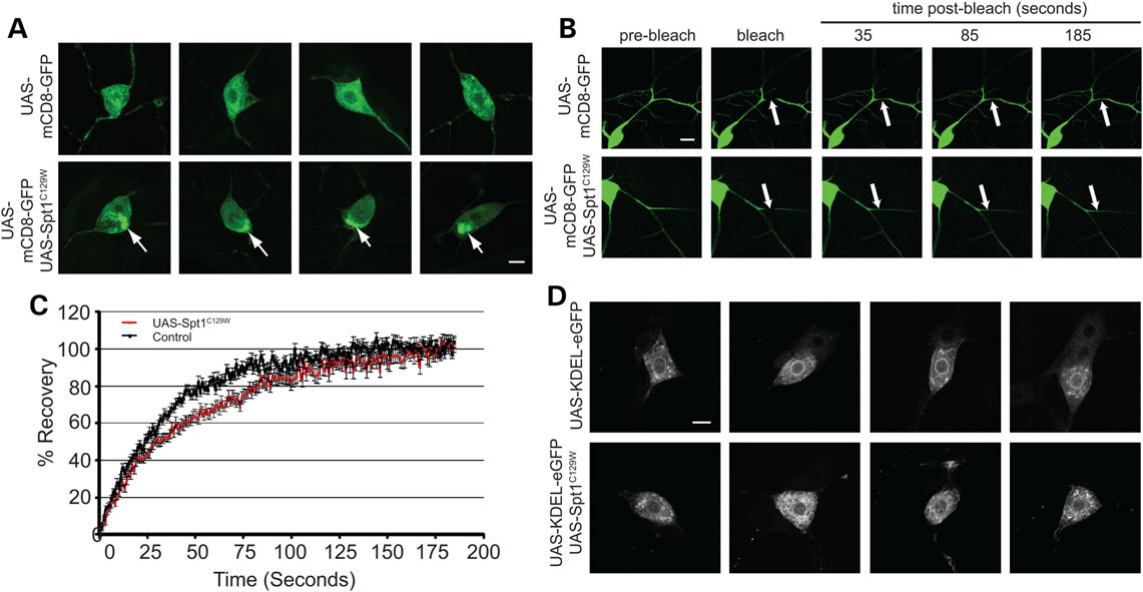
*Spt1^C129W^* expression in class IV da neurons reduces membrane trafficking to the plasma membrane and induces membrane trafficking defects. (**A**) Class IV da neurons expressing the membrane marker mCD8-EGFP and *Spt1^C129W^* develop a perinuclear accumulation of EGFP not observed in wild-type animals. (**B**) FRAP in class IV da neuron secondary dendrites expressing the membrane marker mCD8-EGFP, driven by the *ppk*-GAL4 construct is slower when co-expressing *Spt1^C129W^*. An area of 45 × 15 pixels was bleached using 100% laser power at 488 nm for 200 iterations and monitored for 200 s. t-half for control dendrites is 20 s (*n* = 25) and 31 s for cells expressing SPT1^C129W^ (*n* = 26) with a significant difference of *P* ≤ 0.01. Student's *t*-test, *t* = 2.03, 33 df. (**C**) Examples of fluorescence recovery in wild-type neurons or neurons expressing *Spt1^C129W^*. Scale bar = 10 µm. (**D**) *Spt1^C129W^* expressing neurons show an accumulation of an ER marker (KDEL-EGFP) in the cell body compared with wild-type. Scale bar = 10 µm.

During class IV da arbor morphology analysis, accumulations of mCD8-EGFP fluorescence were observed in neurons expressing *dSpt1^C129W^* that were largely absent in controls (Fig. 4A). It was considered therefore that, under conditions of altered sphingolipid expression, there is reduced trafficking of mCD8-EGFP out of the soma into the plasma membrane of the arbor to alter membrane function. In order to test this theory, fluorescence recovery after photobleaching (FRAP) was performed upon dendrite branches. Figures 4B and C show that FRAP is significantly reduced in class IV da neurons expressing *dSpt1^C129W^* resulting in a *t*^1/2^ (time to half maximal recovery) of 31 s in comparison to 20 s in controls (*P* < 0.01, Student’s *t*-test). Co-expression of an ER marker, Lyz-KDEL-EGFP (33), with *Spt1^C129W^* reveals excessive accumulations of GFP compared with no *Spt1^C129W^* expression (Fig. 4D). These data therefore support the theory that correct sphingolipid function is required for the correct trafficking of mCD8-EGFP into the class IV dendrite arbour and that a secretory deficit is present in neurons expressing d*Spt1^C129W^*.

### *dSpt1^C129W^* or *dSpt1* expression in *Drosophila* larval sensory neurons generates a subtle arborization defect

**Figure 5.**
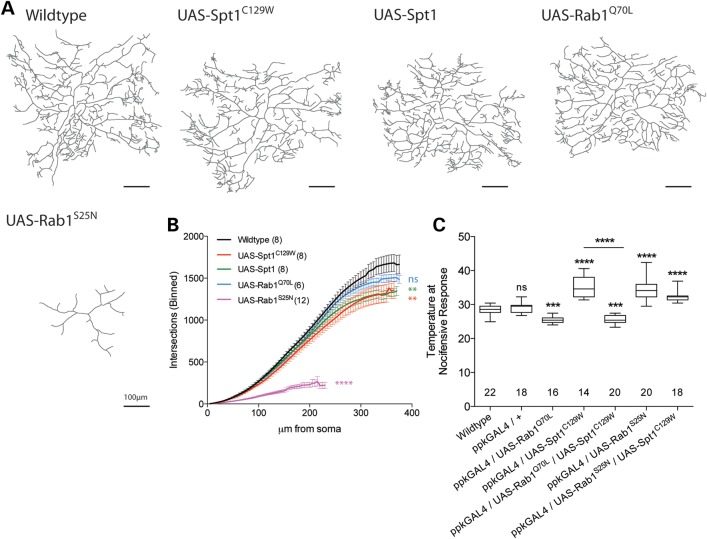
Increased ER–Golgi membrane trafficking rescues *Spt1^C129W^*-induced nociception defects. (**A**) Exhaustive Z-stack confocal images of the v'ada class IV da sensory neurons in larval hemisegment A3 of larvae expressing mCD8-EGFP under control of the *ppk*-GAL4 driver in combination with UAS-*Spt1*, UAS-*Spt1^C129W^*, UAS-*Rab1^Q70L^* or UAS-*Rab1^S25N^*. Scale bar = 10 µm. (**B**) Sholl analysis performed on dendrite traces reveals that *Spt1* or *Spt1^C129W^* expression mildly, yet significantly, reduces arborization. Kolmogorov–Smirnov test, **P < 0.05, *z* = 1.436. (**C**) Co-expression of dominant-active Rab1^Q70L^ significantly rescues *Spt1^C129W^*-expression-induced defective nociception. Expression of *Rab1^Q70L^* increases larval sensitivity to noxious heat, whereas expression of dominant-negative *Rab1^S25N^* causes defective nociception equivalent to that induced by Spt1^C129W^ expression. Co-expression of UAS-Spt1^C129W^ and UAS-Rab1^S25N^ does not significantly worsen the nociceptive defect. ***P ≤ 0.001, ****P ≤ 0.0001, ANOVA, ns = non-significant compared with control.

With respect to the predominant sensory pathology observed in HSAN1, we then expressed *dSpt1* and *dSpt1^C129W^* in the class IV dendritic arborization (da) neurons using the class IV-specific GAL4 driver line *pickpocket-GAL4* (*ppk*-GAL4) (26). Dendritic arbors were visualized using the co-expression of the plasma membrane marker mCD8-EGFP. Figure 5 shows the dendritic arbour of the v'ada neuron, in hemisegment 3 proximal to the location of the muscle 6/7 NMJ. The expression of either *dSpt1* or *dSpt1^C129W^* produces a subtle, yet statistically significant reduction in dendrite arborization, as shown by Sholl analysis (Fig. 5A and B) (27). This subtle alteration in dendritic morphology, at least for the *dSpt1^C129W^* expressing neurons may be due in part to the trafficking deficit of the mCD8-EGFP marker observed in Fig. 4. Together with our data that expression of *dSpt1* causes no nociception defect (Fig. 2), we suggest that the presence of the *dSpt1^C129W^* mutation is sufficient to generate deficits in nociception and that such reductions in function are unlikely to be caused by changes in dendritic morphology.

### Enhancing ER to Golgi traffic rescues *dSpt1^C129W^* expression-induced nociceptive defects

In a genetic screen for mutations affecting sensory dendritic arbour growth, a critical requirement for ER to Golgi trafficking function was identified (34). Identification of mutations in *sec23*, *Sar1* and *Rab1* suggests that ER to Golgi trafficking function is limiting to dendritic growth (34), a finding confirmed in mammalian neurons (35–37). To examine the suggestion that *dSpt1^C129W^* expression may perturb ER to Golgi trafficking affecting sensory function, we introduced a known effector of ER to Golgi membrane trafficking, Rab1. Figure 5C demonstrates that the co-expression of the dominantly active form of *Rab1^Q70L^* completely restores the nociceptive defect observed with *dSpt1^C129W^* expression while expression of the constitutively inactive *Rab1^S25N^* alone phenocopies *dSpt1^C129W^* expression. Co-expression of *Rab1^S25N^* with *dSpt1^C129W^* in Class IV da neurons causes latency in the nociceptive response that is no more severe than either construct alone. Expression of *Rab1^Q70L^* generates a dendritic arbour indistinguishable from wild-type. These data together suggest that sphingolipid depletion, or the presence of DSBs, does indeed induce a trafficking bottle-neck at the ER to Golgi interface and that this can be circumvented via the expression of ER to Golgi trafficking effectors, rescuing nociceptor function.

## Discussion

HSAN1 is characterized by a deficit in peripheral sensory function with ulceration and autonomic disturbances (3–5,38). It is unclear at present why mutations in the globally expressed SPT enzyme should lead to the specific peripheral phenotypes of HSAN1. An initial study suggested the mutation conferred a gain-of-function to the SPT enzyme (7), but later studies in yeast and mammalian cells (13,14) determined an inhibitory function, albeit with normal cellular levels of sphingolipids. A more recently proposed pathological mechanism, the presence of DSBs caused by alanine or glycine incorporation, rather than serine during sphingolipid synthesis suggests a neomorphic mode of action for the *SPT* mutations inducing HSAN1 (15,16) if DSBs are indeed toxic (39,40). These proposed mechanisms are not mutually exclusive, and the data that we present with our *Drosophila* model of HSAN1 supports both.

For confirmation of the dominant loss-of-function mode of action of the *dSpt1^C129W^* mutant SPT subunit, we assayed the development of the larval NMJ. The *lace* locus in *Drosophila* encodes the dSpt2 enzyme (21). The *lace^P^* mutant is a hypomorphic transposon insertion in the *dSpt2* locus that as a homozygote dies early in the pupal stage of development (21) and has been shown to express 2.58% of wild-type sphingolipid levels (25). Figure 1C and D shows that in the *lace* mutant, muscle 6/7 NMJ is severely underdeveloped in terms of bouton number, the observed boutons being larger and fewer than wild-type animals. This is the first description of a sphingolipid deficient synapse and suggests that these complex lipids are essential for regulation of synaptic structure. Similarly, expressing *dSpt1^C129W^* under the control of *Elav*-GAL4 neuronal promoter produces an aberrant developmental phenotype though not as severe. Introduction of *dSpt1* or *dSpt2/lace^P^* heterozygous loss-of-function alleles in the presence of globally or neuronally expressed *dSpt1^C129W^* cause, respectively, early lethality and a synaptic phenotype identical to the *dSpt2/lace^P^* loss-of-function mutant. This phenocopy supports our sphingolipid quantification data shown in Fig. 3, suggesting that *dSpt1^C129W^* expression reduces sphingolipid biogenesis consistent with the early reports of a dominant-negative mutant function (13,14). Reduction of wild-type copy number of *dSpt1* or *dSpt2/lace* subunits of dSpt in the presence of the *dSpt1^C129W^* mutant induces a severe phenotype identical to a strong *dSpt2* hypomorphic mutation with a very low sphingolipid content. These genetic combinations confirm the potential dominant-negative mode of action for the disease mutation in a peripheral neuron in our system.

Expression of *dSpt1^C129W^* in the class IV md sensory neurons responsible for thermo- and mechanosensory function in *Drosophila* larvae (28,41,42) induces a mild morphological reduction in dendrite terminals consistent with loss of epidermal innervation in *SPT1^C133W/Y^* carrying HSAN1 patients (5,43). These larvae also display a deficit in thermosensation, showing a latency to respond to rising temperature consistent with a lack of function in the class IV md neurons. Interestingly, optogenetic stimulation of the class IV md neurons in the presence of the dSpt1^C129W^ mutant can still elicit a nocifensive response (Supplementary Material, Movie S1) indicating some synaptic function remains in these compromised neurons. Synaptic structure of Class IV da neurons in the ventral nerve cord is preserved when dSpt1^C129W^ is expressed (Supplementary Material, Fig. S2). These data suggest that the functional deficit may not centre on synaptic function in dSpt1^C129W^ compromised sensory neurons but may lie in the dendritic arbour.

On examination of larval sensory neurons expressing *dSpt1^C129W^*, we observed a membrane trafficking deficit in cell soma. The data presented here, an expression of constitutively active Rab1 rescue of a dSpt1^C129W^-induced sensory deficit, suggests that *dSpt1^C129W^* expression induces an ER to Golgi trafficking restriction and that this disrupts nociception. Recent reports suggest a novel activity for the SPT enzyme bearing the mutated subunit. The C133W mutation is proposed to confer a preferred specificity for alanine over serine in the condensation reaction between serine and palmitoyl-CoA (15,16) to generate DSBs, a finding confirmed in this study with the alanine fed dSpt1^C129W^ expressing animal. In this scheme, it is proposed that the DSBs generate stress in the ER (16) and defects in neurite outgrowth and retraction (15). Membrane biogenesis is critically dependent on the orchestration between lipid and protein synthesis at the ER. Export of protein complexes from the ER can be dependent on the availability of ceramide (44), while the export of GPI-anchored proteins are critically dependent on the presence of sphingolipids and sterols (45,46). Disruption of the ORM1/2 proteins, important regulators of SPT function, leads to severe defects in ER function with a slowed ER-to-Golgi transport (47). Dendrites are particularly rich in components of the secretory apparatus with ER export sites and Golgi outposts assembled regularly throughout the dendritic tree (48–50). A genetic screen in *Drosophila* larvae, using the growth of the class IV md neurons as a model identified factors of ER-to-Golgi traffic as essential for proper dendritic growth (34). Studies in mammalian growing neurites confirm this requirement (35,36), suggesting a critical reliance in dendrites for secretory function. ER export sites are essential for dendrite outgrowth, but they are also observed to recruit neurotransmitter receptors upon metabotropic activation and neuronal activity reflecting a potential role in regulating dendritic function (35,48). Recent identification of dominant mutations in Atlastin1 and 3, proteins required for homotypic membrane fusion in the ER, in HSAN1 patients (51–54) similarly point to ER function as a ‘pinch-point’ in distal peripheral sensory function.

Oral supplementation of l-serine to the diet of mSPT1^C133W^ expressing mice decreased DSB levels in plasma, paralleling similar observations in *SPT1^C133W^* carrying HSAN1 patients (55). We observe a similar effect in our *Drosophila* model system with alanine feeding to dSpt1^C129W^ expressing animals inducing lethality that can be partially rescued by supplementation of l-serine in the food source. We also find that we can rescue synaptic structure in an *Elav*-GAL4/UAS-*dSpt^C129W^*, *lace^P^* animal by feeding serine in the diet suggesting that the potential dominant-negative action of dSpt^C129W^ may be due to incorporation of alanine in nascent sphingolipids.

We have demonstrated that the causative mutation of HSAN1, *SPT1^C133W^* when expressed in *Drosophila* (as dSpt1^C129W^) acts dominantly to reduce sphingolipid synthesis, generate synthesis of DSBs and perturb the development of dendrites, neuromuscular synapses and nociceptive function of the class IV da sensory neurons. This reveals an essential function of sphingolipids in the development of these neurons *in vivo*. We also demonstrate that the expression of dSpt1^C129W^ causes a significant reduction in the sensitivity of third instar larvae to noxious heat stimuli, producing a phenotype consistent with that observed in HSAN1 patients. Our data reveal that this sensory dysfunction may be due to an aberrant trafficking bottle-neck in sensory dendrites, consistent with dysfunction in the ER. Thus we present a novel mechanism for the pathology observed in HSAN1 due to mutations in SPT1 in a simple model system amenable that recapitulates many aspects of the human disease.

## Materials and Methods

### *Drosophila* stocks and husbandry

*Drosophila* maintenance and crosses were performed using standard yeast–agar media at 25°C. Stocks used in this study: UAS-d*Spt1^C129W^*, UAS-*Spt1* (this study), UAS-*ChRh2* (56) (Gero Miesenböck, Oxford), UAS-*LYZ-GFP-KDEL* (33) (Mary Lilly, NIH, Bethesda), UAS-Tetanus-toxin (29), *pain^1^* (28) (Dan Tracey, Durham, NC), *lace^P^* (21) (John Roote, Cambridge), *SPT1^l(2)SH1626^* (19) (Stephen Hou, NIH, Bethesda), *pickpocket-*GAL4 (26) (Wes Grueber, Columbia). The following stocks were obtained from the Bloomington Stock Centre, Indiana: *w^1118^* (used throughout as wild-type), *Df(2R)Vg-B*, *Elav*-GAL4 *tubulin*-GAL4, UAS-*mCD8-GFP*, UAS-*YFP-Rab1Q70L*, UAS-*YFP-Rab1S25N* (57), *l(2)49Fb^1^*, *l(2)49Fb^4^* (20).

### Generation of UAS-*dSpt1* and UAS-d*Spt1^C129W^* transgenes

The C129W mutation was introduced into the *dSpt1* cDNA (RE58623) by site-directed-mutagenesis using the following forward (CGGAGTTGGATCTTGGGGACCTCGG) and reverse (CCGAGGTCCCCAAGATCCAACCTCCG) primers. Each construct was then sequence-verified and cloned into pUASt (22). Microinjection into *w-* embryos was then carried out using standard procedures.

### NMJ analysis

Third instar wandering larvae were dissected in PBS, fixed in 3.7% formaldehyde/PBS for 7 min and stained using the appropriate primary antibodies, Cy3 conjugated goat anti-HRP (1:200, Stratech scientific); rabbit anti-synaptotagmin (1:2000) (58). Synaptic bouton numbers at muscles 6/7, hemisegment A3, were determined by counting each distinct, spherical, anti-synaptotagmin positive varicosity contacting the muscle. As synaptic bouton number has been shown to increase proportionally with muscle surface area (MSA) synaptic bouton numbers were normalized against MSA by dividing the bouton number by the MSA and multiplying by mean wild-type MSA as described by (59). Muscles and synapses were imaged using an AxioCam HRC camera on a Axiovert 200 invert fluorescence microscope using Plan-NeoFluar 10×/0.3 and 40×/0.75 lenses, with Axiovision Rel. 4.8 software. Measurements were made from images using ImageJ.

### Dendritic arbour morphology

Third instar larvae were dissected, and mounted. Dendritic field imaging was performed using a CarlZeiss Axiovert 200M inverted confocal microscope with a 20× objective lens. High power excitation wavelength (488 nm) light and exhaustive Z-stack conditions were used to image the v’ ada class IV da neuron. Neurons were traced using NeuronJ imageJ plugin (60). Sholl analysis (27) was then performed upon these tracings using the Sholl analysis ImageJ plugin (62). Cumulative branch distribution data were interrogated using the Kolmogorov–Smirnov test, *n* > 5.

### Sphingolipid quantification

Larvae were rapidly dissected in cold PBS to remove the fat bodies and subsequently frozen. After homogenization 1 mg was taken and used for each extraction. Total sphingolipids were extracted overnight in chloroform:methanol (1:1), and sample cleanup, labelling with *o*-phthalaldehyde and analysis by HPLC were as described previously (61). Standards of C14 and C16 sphingoid bases were from Matreya, C18 sphingoid bases were from Sigma and an internal control C20 sphingosine was from Avanti Polar Lipids.

### Larval nociception analysis

Third instar nociception assay based upon nocifensive characteristic escape behaviours to heat (17,28). Wandering third instar larvae were placed into a 30-μl water droplet on a petri dish. The petri dish was then placed onto a standard heater block set to 70°C. The time taken to perform escape behaviour was recorded, with respect to the first violent head-swing. Droplet temperature calibration was measured using a thermocouple with a digital read-out (Tenma 72-2060, Tenma Test Equipment Ohio, USA), held into the water droplet (*n* > 20). Nociception data interrogated using one-way ANOVA (*n* > 15). Nociception movies were recorded using a standard WebCam suspended above the heater block.

### Secondary dendrite fluorescence resonance analysis after photobleaching imaging

Live larval dissection was performed as described for NMJ analysis above with the exception that experiments were performed in physiological saline. Specifically, wandering third-instar larvae were selected and were recorded in HL3 saline (70 mm NaCl, 5 mm KCl, 10 mm NaHCO_3_, 115 mm sucrose, 5 mm trehalose and 5 mm HEPES) with 0.4 mm Ca^2+^. Class IV da neurons were located using CarlZeiss Axiovert 200M inverted confocal microscope with a 63× objective lens. The 100% laser power (488 nm, 20 mW) for 200 iterations was used to bleach region of interest 1 (ROI1) followed by 200 s post-bleach imaging to observe recovery. Acquisitional bleaching was controlled for using a second ROI and the following equation ((*ROI1*/*ROI2*)/(*t* = *0ROI1*/*t* = *0ROI2*)) *100 = % recovery (*t* = 0, pre-bleach fluorescence) (*n* > 25). High magnification images of class IV da neuron cell bodies and muscle 6/7 NMJs were made using a CarlZeiss Axiovert 200M inverted confocal microscope with a 63× objective lens.

### Alanine and serine feeding

*Drosophila* instant food (Carolina Biological Supply) was reconstituted using distilled water supplemented with l-serine or l-alanine. *Drosophila* crosses were added directly to this food source and allowed to lay for 3 days. Resultant offspring were counted and noted for genetic markers to assess survival. Crosses used were: (1) wild-type – *w^1118^*, (2) *tubulin*-GAL4/*TM6b, Hu, Tb*x UAS-*dSpt1^C129W^* and (3) *tubulin*-GAL4/*TM6b, Hu, Tb x UAS-dSpt1*.

## Supplementary Material

Supplementary Material is available at *HMG* online.

## Funding

This work was funded by a Quota studentship from the MRC (to M.C.W.O.) and a Medical Research Council grant (G0400580 to S.T.S.). Work in the E.L.-E. lab was supported by an RCUK Fellowship (to E.L.-E.), Sport Aiding Medical Research for Kids and a project grant from the Royal Society.

## Supplementary Material

Supplementary Data

## References

[1] Tocantins L.M., Reimann H.A. (1939) Perforating ulcers of feet, with osseus atrophy: in a family with other evidences of dysgenesis (harelip, cleft palate): an instance of probable myelodysplasia. JAMA, 112, 2251–2255.

[2] van Bogaert L. (1957) Familial ulcers, mutilating lesions of the extremities, and acro-osteolysis. Br. Med. J., 2, 367–371. 13446485 10.1136/bmj.2.5041.367 PMC1962095

[3] Denny-Brown D. (1951) Hereditary sensory radicular neuropathy. J. Neurol. Neurosurg. Psychiatr., 14, 237–252. 14898294 10.1136/jnnp.14.4.237 PMC499526

[4] Auer-Grumbach M., De Jonghe P., Verhoeven K., Timmerman V., Wagner K., Hartung H.-P., Nicholson G.A. (2003) Autosomal dominant inherited neuropathies with prominent sensory loss and mutilations: a review. Arch. Neurol., 60, 329–334. 12633143 10.1001/archneur.60.3.329

[5] Houlden H., King R., Blake J., Groves M., Love S., Woodward C., Hammans S., Nicoll J., Lennox G., O'Donovan D.G. et al (2006) Clinical, pathological and genetic characterization of hereditary sensory and autonomic neuropathy type 1 (HSAN I). Brain, 129, 411–425. 16364956 10.1093/brain/awh712

[6] Bejaoui K., Wu C., Scheffler M.D., Haan G., Ashby P., Wu L., de Jong P., Brown R.H. (2001) SPTLC1 is mutated in hereditary sensory neuropathy, type 1. Nat. Genet., 27, 261–262. 11242106 10.1038/85817

[7] Dawkins J.L., Hulme D.J., Brahmbhatt S.B., Auer-Grumbach M., Nicholson G.A. (2001) Mutations in SPTLC1, encoding serine palmitoyltransferase, long chain base subunit-1, cause hereditary sensory neuropathy type I. Nat. Genet., 27, 309–312. 11242114 10.1038/85879

[8] Yard B.A., Carter L.G., Johnson K.A., Overton I.M., Dorward M., Liu H., McMahon S.A., Oke M., Puech D., Barton G.J. et al (2007) The structure of serine palmitoyltransferase; gateway to sphingolipid biosynthesis. J. Mol. Biol., 370, 870–886. 17559874 10.1016/j.jmb.2007.04.086

[9] Hornemann T., Penno A., Rütti M.F., Ernst D., Kivrak-Pfiffner F., Rohrer L., Eckardstein von A. (2009) The SPTLC3 subunit of serine palmitoyltransferase generates short chain sphingoid bases. J. Biol. Chem., 284, 26322–26330. 19648650 10.1074/jbc.M109.023192 PMC2785320

[10] Rotthier A., Auer-Grumbach M., Janssens K., Baets J., Penno A., Almeida-Souza L., Van Hoof K., Jacobs A., De Vriendt E., Schlotter-Weigel B. et al (2010) Mutations in the SPTLC2 subunit of serine palmitoyltransferase cause hereditary sensory and autonomic neuropathy type I. Am. J. Hum. Genet., 87, 513–522. 20920666 10.1016/j.ajhg.2010.09.010 PMC2948807

[11] Murphy S.M., Ernst D., Wei Y., Laurá M., Liu Y.-T., Polke J., Blake J., Winer J., Houlden H., Hornemann T. et al (2013) Hereditary sensory and autonomic neuropathy type 1 (HSANI) caused by a novel mutation in SPTLC2. Neurology, 80, 2106–2111. 23658386 10.1212/WNL.0b013e318295d789 PMC3716354

[12] Ikushiro H., Hayashi H., Kagamiyama H. (2001) A water-soluble homodimeric serine palmitoyltransferase from *Sphingomonas paucimobilis* EY2395T strain. Purification, characterization, cloning, and overproduction. J. Biol. Chem., 276, 18249–18256. 11279212 10.1074/jbc.M101550200

[13] Dedov V.N., Dedova I.V., Merrill A.H., Nicholson G.A. (2004) Activity of partially inhibited serine palmitoyltransferase is sufficient for normal sphingolipid metabolism and viability of HSN1 patient cells. Biochim. Biophys. Acta, 1688, 168–175. 14990347 10.1016/j.bbadis.2003.12.005

[14] McCampbell A., Truong D., Broom D.C., Allchorne A., Gable K., Cutler R.G., Mattson M.P., Woolf C.J., Frosch M.P., Harmon J.M. et al (2005) Mutant SPTLC1 dominantly inhibits serine palmitoyltransferase activity in vivo and confers an age-dependent neuropathy. Hum. Mol. Genet., 14, 3507–3521. 16210380 10.1093/hmg/ddi380

[15] Penno A., Reilly M.M., Houlden H., Laurá M., Rentsch K., Niederkofler V., Stoeckli E.T., Nicholson G., Eichler F., Brown R.H. et al (2010) Hereditary sensory neuropathy type 1 is caused by the accumulation of two neurotoxic sphingolipids. J. Biol. Chem., 285, 11178–11187. 20097765 10.1074/jbc.M109.092973 PMC2856995

[16] Gable K., Gupta S.D., Han G., Niranjanakumari S., Harmon J.M., Dunn T.M. (2010) A disease-causing mutation in the active site of serine palmitoyltransferase causes catalytic promiscuity. J. Biol. Chem., 285, 22846–22852. 20504773 10.1074/jbc.M110.122259 PMC2906276

[17] Oswald M., Rymarczyk B., Chatters A., Sweeney S.T. (2011) A novel thermosensitive escape behavior in Drosophila larvae. Fly (Austin), 5, 304–306. 21914977 10.4161/fly.5.4.17810 PMC3266071

[18] Yasuda S., Nishijima M., Hanada K. (2003) Localization, topology, and function of the LCB1 subunit of serine palmitoyltransferase in mammalian cells. J. Biol. Chem., 278, 4176–4183. 12464627 10.1074/jbc.M209602200

[19] Oh S.-W., Kingsley T., Shin H.-H., Zheng Z., Chen H.-W., Chen X., Wang H., Ruan P., Moody M., Hou S.X. (2003) A P-element insertion screen identified mutations in 455 novel essential genes in Drosophila. Genetics, 163, 195–201. 12586707 10.1093/genetics/163.1.195 PMC1462436

[20] Lasko P.F., Pardue M.L. (1988) Studies of the genetic organization of the vestigial microregion of *Drosophila melanogaster*. Genetics, 120, 495–502. 3143621 10.1093/genetics/120.2.495 PMC1203527

[21] Adachi-Yamada T., Gotoh T., Sugimura I., Tateno M., Nishida Y., Onuki T., Date H. (1999) De novo synthesis of sphingolipids is required for cell survival by down-regulating c-Jun N-terminal kinase in Drosophila imaginal discs. Mol. Cell. Biol., 19, 7276–7286. 10490662 10.1128/mcb.19.10.7276 PMC84720

[22] Brand A.H., Perrimon N. (1993) Targeted gene expression as a means of altering cell fates and generating dominant phenotypes. Development, 118, 401–415. 8223268 10.1242/dev.118.2.401

[23] Collins C.A., DiAntonio A. (2007) Synaptic development: insights from Drosophila. Curr. Opin. Neurobiol., 17, 35–42. 17229568 10.1016/j.conb.2007.01.001

[24] Ruiz-Cañada C., Budnik V. (2006) Introduction on the use of the Drosophila embryonic/larval neuromuscular junction as a model system to study synapse development and function, and a brief summary of pathfinding and target recognition. Int. Rev. Neurobiol., 75, 1–31. 17137921 10.1016/S0074-7742(06)75001-2

[25] Fyrst H., Herr D.R., Harris G.L., Saba J.D. (2004) Characterization of free endogenous C14 and C16 sphingoid bases from Drosophila melanogaster. J. Lipid Res., 45, 54–62. 13130120 10.1194/jlr.M300005-JLR200

[26] Grueber W.B., Ye B., Moore A.W., Jan L.Y., Jan Y.N. (2003) Dendrites of distinct classes of Drosophila sensory neurons show different capacities for homotypic repulsion. Curr. Biol., 13, 618–626. 12699617 10.1016/s0960-9822(03)00207-0

[27] Sholl D.A. (1953) Dendritic organization in the neurons of the visual and motor cortices of the cat. J. Anat., 87, 387–406. 13117757 PMC1244622

[28] Tracey W.D., Wilson R.I., Laurent G., Benzer S. (2003) painless, a Drosophila gene essential for nociception. Cell, 113, 261–273. 12705873 10.1016/s0092-8674(03)00272-1

[29] Sweeney S.T., Broadie K., Keane J., Niemann H., O'Kane C.J. (1995) Targeted expression of tetanus toxin light chain in Drosophila specifically eliminates synaptic transmission and causes behavioral defects. Neuron, 14, 341–351. 7857643 10.1016/0896-6273(95)90290-2

[30] Neely G.G., Keene A.C., Duchek P., Chang E.C., Wang Q.-P., Aksoy Y.A., Rosenzweig M., Costigan M., Woolf C.J., Garrity P.A. et al (2011) TrpA1 regulates thermal nociception in Drosophila. PLoS One, 6, e24343. 21909389 10.1371/journal.pone.0024343 PMC3164203

[31] Lima S.Q., Miesenböck G. (2005) Remote control of behavior through genetically targeted photostimulation of neurons. Cell, 121, 141–152. 15820685 10.1016/j.cell.2005.02.004

[32] Zhang W., Ge W., Wang Z. (2007) A toolbox for light control of Drosophila behaviors through Channelrhodopsin 2-mediated photoactivation of targeted neurons. Eur. J. Neurosci., 26, 2405–2416. 17970730 10.1111/j.1460-9568.2007.05862.x

[33] Snapp E.L., Iida T., Frescas D., Lippincott-Schwartz J., Lilly M.A. (2004) The fusome mediates intercellular endoplasmic reticulum connectivity in Drosophila ovarian cysts. Mol. Biol. Cell, 15, 4512–4521. 15292454 10.1091/mbc.E04-06-0475 PMC519145

[34] Ye B., Zhang Y., Song W., Younger S.H., Jan L.Y., Jan Y.N. (2007) Growing dendrites and axons differ in their reliance on the secretory pathway. Cell, 130, 717–729. 17719548 10.1016/j.cell.2007.06.032 PMC2020851

[35] Aridor M., Fish K.N. (2009) Selective targeting of ER exit sites supports axon development. Traffic, 10, 1669–1684. 19761544 10.1111/j.1600-0854.2009.00974.x PMC2763039

[36] Horton A.C., Rácz B., Monson E.E., Lin A.L., Weinberg R.J., Ehlers M.D. (2005) Polarized secretory trafficking directs cargo for asymmetric dendrite growth and morphogenesis. Neuron, 48, 757–771. 16337914 10.1016/j.neuron.2005.11.005

[37] Horton A.C., Ehlers M.D. (2004) Secretory trafficking in neuronal dendrites. Nat. Cell Biol., 6, 585–591. 15232591 10.1038/ncb0704-585

[38] Thomas P.K. (1993) Hereditary sensory neuropathies. Brain Pathol., 3, 157–163. 8293177 10.1111/j.1750-3639.1993.tb00740.x

[39] Duan J., Merrill A.H. (2015) 1-Deoxysphingolipids encountered exogenously and made de novo: dangerous mysteries inside an enigma. J. Biol. Chem., 290, 15380–15389. 25947379 10.1074/jbc.R115.658823 PMC4505451

[40] Bertea M., Rütti M.F., Othman A., Marti-Jaun J., Hersberger M., Eckardstein von A., Hornemann T. (2010) Deoxysphingoid bases as plasma markers in diabetes mellitus. Lipids Health Dis., 9, 84. 20712864 10.1186/1476-511X-9-84 PMC2931514

[41] Hwang R.Y., Stearns N.A., Tracey W.D. (2012) The ankyrin repeat domain of the TRPA protein painless is important for thermal nociception but not mechanical nociception. PLoS One, 7, e30090. 22295071 10.1371/journal.pone.0030090 PMC3266251

[42] Zhong L., Bellemer A., Yan H., Ken H., Jessica R., Hwang R.Y., Pitt G.S., Tracey W.D. (2012) Thermosensory and nonthermosensory isoforms of *Drosophila melanogaster* TRPA1 reveal heat-sensor domains of a thermoTRP channel. Cell Rep., 1, 43–55. 22347718 10.1016/j.celrep.2011.11.002 PMC3278078

[43] Fridman V., Oaklander A.L., David W.S., Johnson E.A., Pan J., Novak P., Brown R.H., Eichler F.S. (2015) Natural history and biomarkers in hereditary sensory neuropathy type 1. Muscle Nerve, 51, 489–495. 25042817 10.1002/mus.24336 PMC4484799

[44] Lee M.C.S., Hamamoto S., Schekman R. (2002) Ceramide biosynthesis is required for the formation of the oligomeric H+-ATPase Pma1p in the yeast endoplasmic reticulum. J. Biol. Chem., 277, 22395–22401. 11950838 10.1074/jbc.M200450200

[45] Watanabe R., Funato K., Venkataraman K., Futerman A.H., Riezman H. (2002) Sphingolipids are required for the stable membrane association of glycosylphosphatidylinositol-anchored proteins in yeast. J. Biol. Chem., 277, 49538–49544. 12393888 10.1074/jbc.M206209200

[46] Runz H., Miura K., Weiss M., Pepperkok R. (2006) Sterols regulate ER-export dynamics of secretory cargo protein ts-O45-G. EMBO J., 25, 2953–2965. 16794576 10.1038/sj.emboj.7601205 PMC1500972

[47] Han S., Lone M.A., Schneiter R., Chang A. (2010) Orm1 and Orm2 are conserved endoplasmic reticulum membrane proteins regulating lipid homeostasis and protein quality control. Proc. Natl. Acad. Sci. USA, 107, 5851–5856. 20212121 10.1073/pnas.0911617107 PMC2851911

[48] Aridor M., Guzik A.K., Bielli A., Fish K.N. (2004) Endoplasmic reticulum export site formation and function in dendrites. J. Neurosci., 24, 3770–3776. 15084657 10.1523/JNEUROSCI.4775-03.2004 PMC6729346

[49] Gardiol A., Racca C., Triller A. (1999) Dendritic and postsynaptic protein synthetic machinery. J. Neurosci., 19, 168–179. 9870948 10.1523/JNEUROSCI.19-01-00168.1999 PMC6782360

[50] Horton A.C., Ehlers M.D. (2003) Dual modes of endoplasmic reticulum-to-Golgi transport in dendrites revealed by live-cell imaging. J. Neurosci., 23, 6188–6199. 12867502 10.1523/JNEUROSCI.23-15-06188.2003 PMC6740539

[51] Kornak U., Mademan I., Schinke M., Voigt M., Krawitz P., Hecht J., Barvencik F., Schinke T., Gießelmann S., Beil F.T. et al (2014) Sensory neuropathy with bone destruction due to a mutation in the membrane-shaping atlastin GTPase 3. Brain, 137, 683–692. 24459106 10.1093/brain/awt357

[52] Guelly C., Zhu P.-P., Leonardis L., Papić L., Zidar J., Schabhüttl M., Strohmaier H., Weis J., Strom T.M., Baets J. et al (2011) Targeted high-throughput sequencing identifies mutations in atlastin-1 as a cause of hereditary sensory neuropathy type I. Am. J. Hum. Genet., 88, 99–105. 21194679 10.1016/j.ajhg.2010.12.003 PMC3014370

[53] Leonardis L., Auer-Grumbach M., Papić L., Zidar J. (2012) The N355 K atlastin 1 mutation is associated with hereditary sensory neuropathy and pyramidal tract features. Eur. J. Neurol., 19, 992–998. 22340599 10.1111/j.1468-1331.2012.03665.x

[54] Fischer D., Schabhüttl M., Wieland T., Windhager R., Strom T.M., Auer-Grumbach M. (2014) A novel missense mutation confirms ATL3 as a gene for hereditary sensory neuropathy type 1. Brain, 137, e286. 24736309 10.1093/brain/awu091

[55] Garofalo K., Penno A., Schmidt B.P., Lee H.-J., Frosch M.P., Eckardstein von A., Brown R.H., Hornemann T., Eichler F.S. (2011) Oral l-serine supplementation reduces production of neurotoxic deoxysphingolipids in mice and humans with hereditary sensory autonomic neuropathy type 1. J. Clin. Invest., 121, 4735–4745. 22045570 10.1172/JCI57549 PMC3225995

[56] Schroll C., Riemensperger T., Bucher D., Ehmer J., Völler T., Erbguth K., Gerber B., Hendel T., Nagel G., Buchner E. et al (2006) Light-induced activation of distinct modulatory neurons triggers appetitive or aversive learning in Drosophila larvae. Curr. Biol., 16, 1741–1747. 16950113 10.1016/j.cub.2006.07.023

[57] Zhang J., Schulze K.L., Hiesinger P.R., Suyama K., Wang S., Fish M., Acar M., Hoskins R.A., Bellen H.J., Scott M.P. (2007) Thirty-one flavors of Drosophila rab proteins. Genetics, 176, 1307–1322. 17409086 10.1534/genetics.106.066761 PMC1894592

[58] West R.J.H., Lu Y., Marie B., Gao F.-B., Sweeney S.T. (2015) Rab8, POSH, and TAK1 regulate synaptic growth in a Drosophila model of frontotemporal dementia. J. Cell Biol., 208, 931–947. 25800055 10.1083/jcb.201404066 PMC4384727

[59] Milton V.J., Jarrett H.E., Gowers K., Chalak S., Briggs L., Robinson I.M., Sweeney S.T. (2011) Oxidative stress induces overgrowth of the Drosophila neuromuscular junction. Proc. Natl. Acad. Sci. USA, 108, 17521–17526. 21987827 10.1073/pnas.1014511108 PMC3198377

[60] Meijering E., Jacob M., Sarria J.-C.F., Steiner P., Hirling H., Unser M. (2004) Design and validation of a tool for neurite tracing and analysis in fluorescence microscopy images. Cytometry A, 58, 167–176. 15057970 10.1002/cyto.a.20022

[61] Lloyd-Evans E., Morgan A.J., He X., Smith D.A., Elliot-Smith E., Sillence D.J., Churchill G.C., Schuchman E.H., Galione A., Platt F.M. (2008) Niemann-Pick disease type C1 is a sphingosine storage disease that causes deregulation of lysosomal calcium. Nat. Med., 14, 1247–1255. 18953351 10.1038/nm.1876

[62] Ferreira T.A., Blackman A.V., Oyrer J., Jayabal S., Chung A.J., Watt A.J., Sjöström P.J., van Meyel D.J. (2014) Neuronal morphology directly from bitmap images. Nat. Methods., 11, 982–984. 25264773 10.1038/nmeth.3125 PMC5271921

