# Supplementary material for: Identification of dietary alanine toxicity and trafficking dysfunction in a Drosophila model of hereditary sensory and autonomic neuropathy type 1
Source: Hum Mol Genet. 2015 Sep 22;24(24):6899–909. doi: 10.1093/hmg/ddv390 (PMC4654049; doi:10.1093/hmg/ddv390)
Supplement: Supplementary Data [file supp_24_24_6899__index.html]

Identification of Dietary Alanine Toxicity and Trafficking Dysfunction in a Drosophila Model of Hereditary Sensory and Autonomic Neuropathy type1 (HSAN1) — Identification of dietary alanine toxicity and trafficking dysfunction in a Drosophila model of hereditary sensory and autonomic neuropathy type 1 — Identification of dietary alanine toxicity and trafficking dysfunction in a Drosophila model of hereditary sensory and autonomic neuropathy type 1 — Supplementary Data 

# Identification of dietary alanine toxicity and trafficking dysfunction in a *Drosophila* model of hereditary sensory and autonomic neuropathy type 1

## Supplementary Data

Supplementary Data

- Supplementary Data - Docx file
- Supplementary movie1 - MOV file
