# Supplementary material for: Identification of dietary alanine toxicity and trafficking dysfunction in a Drosophila model of hereditary sensory and autonomic neuropathy type 1
Source: Hum Mol Genet. 2015 Sep 22;24(24):6899–909. doi: 10.1093/hmg/ddv390 (PMC4654049; doi:10.1093/hmg/ddv390)
Supplement: Supplementary Data [file supp_ddv390_ddv390supp.docx]

**Supplementary Methods**

**Light-Activated Nociception**

3^rd^ instar larvae expressing UAS-*channelrhodopsin-2* (Lima and Miesenböck, 2005) and UAS-*Spt1^C129W^* under control of the ppk-GAL4 driver in the classIV da neurons were placed into a 30μl water droplet on a petri dish. Larval behaviour was then recorded using a CarlZiess NeoLumar fluorescence dissecting microscope illuminated by a remote swan-neck halogen light. Larvae were imaged under white light for 15 seconds, following which blue-light (as would be used to image GFP and stimulate channelrhodopsin2) was exposed to the droplet and the response recorded.


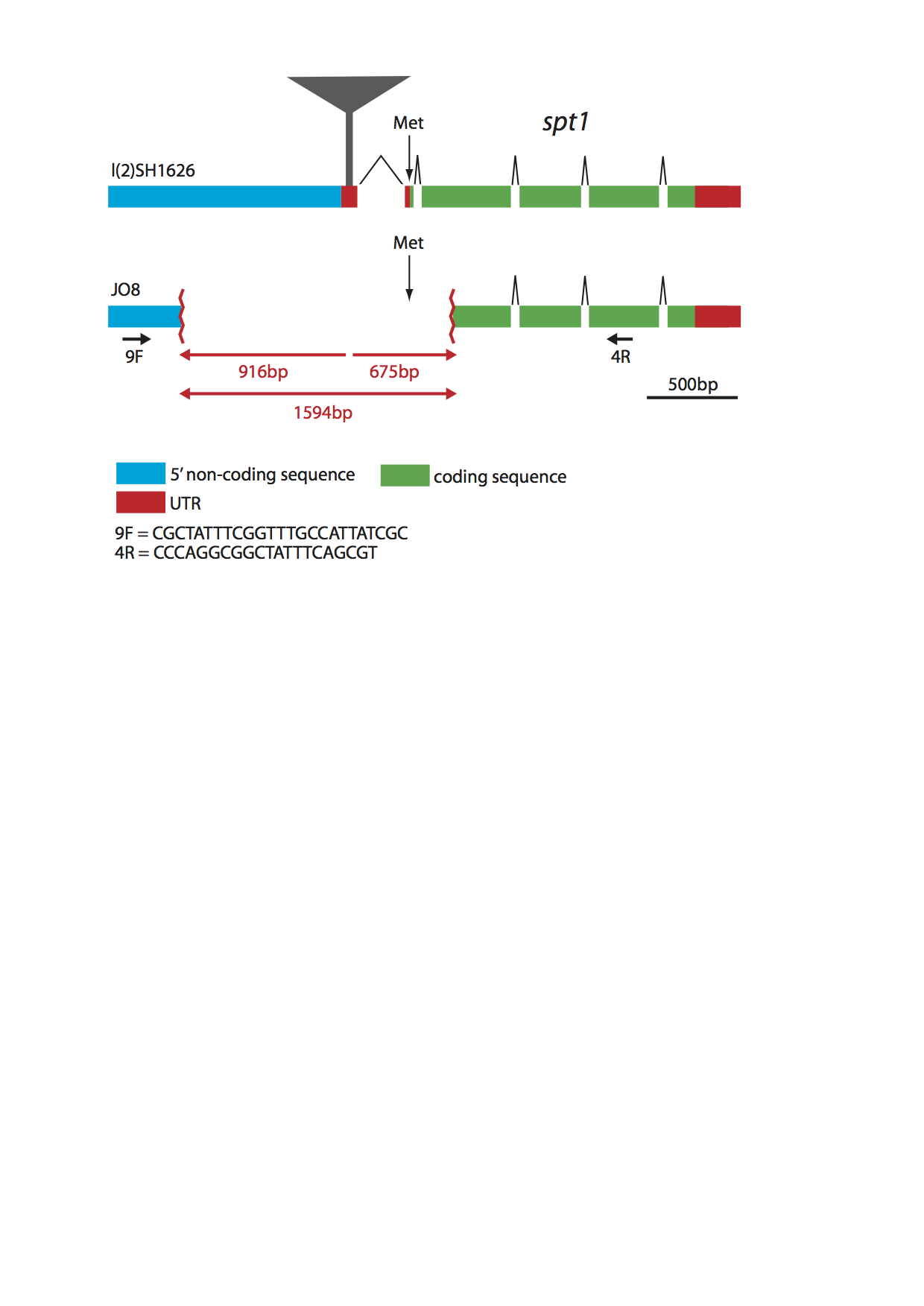


**SFigure 1. Map of the Spt1 locus and generation of *Spt1^JO8^* deletion allele.** Oligonucelotide sequences at bottom left used to confirm the *P*-element imprecise excision induced deletion allele *Spt1^J08^*. Original P-element insertion used to generate the deletion is the *dSpt1^l(2)SH1626^* stock (19).

**
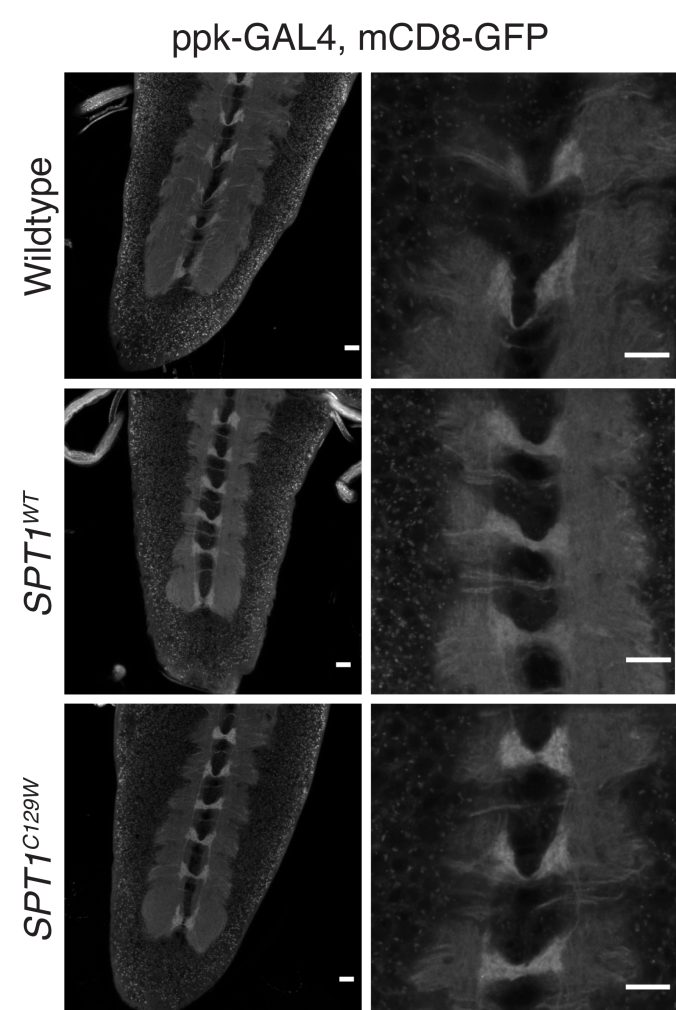
SFigure 2. Expression of *Spt1* or *Spt1^C129W^* does not disturb the general arrangement of the Class IV neuronal synapses in the ventral nerve cord.** Images of larval ventral nerve cords from animals expressing UAS-*mCD8-EGFP* under control of the *ppk*-GAL4 driver, or co-expressing either UAS-*Spt1* or UAS-*Spt1^C129W^*. Scale bars = 10µm

**SMovie: Optogenetic activation of dSpt1^C129W^ compromised sensory neurons.** To confirm that the classIV da neurons contribute to nociceptive escape behaviour, UAS-*channel-rhodopsin2* (UAS-*ChRh2*,(31)) was expressed using *ppk*-GAL4 in the presence of UAS-*Spt1^C129W^*. UAS-ChRh2 non-specifically gates cations in response to blue light, similar to that used routinely to excite GFP. It has also been reported that UAS-*ChRh2* expression in the class IV da neurons is able to produce escape behaviour upon blue-light activation (32). Movie demonstrates that stimulating the classIV da neurons only, is sufficient to elicit an escape response, confirming that the classIV neurons contribute to this behaviour.
